# Supplementary material for: AUX1-mediated root hair auxin influx governs SCFTIR1/AFB-type Ca2+ signaling
Source: Nat Commun. 2018 Mar 21;9:1174. doi: 10.1038/s41467-018-03582-5 (PMC5862985; doi:10.1038/s41467-018-03582-5)
Supplement: Supplementary file 1 — Supplementary Information(PDF 883 kb) [file 41467_2018_3582_MOESM1_ESM.pdf]

Supplementary Figure 1

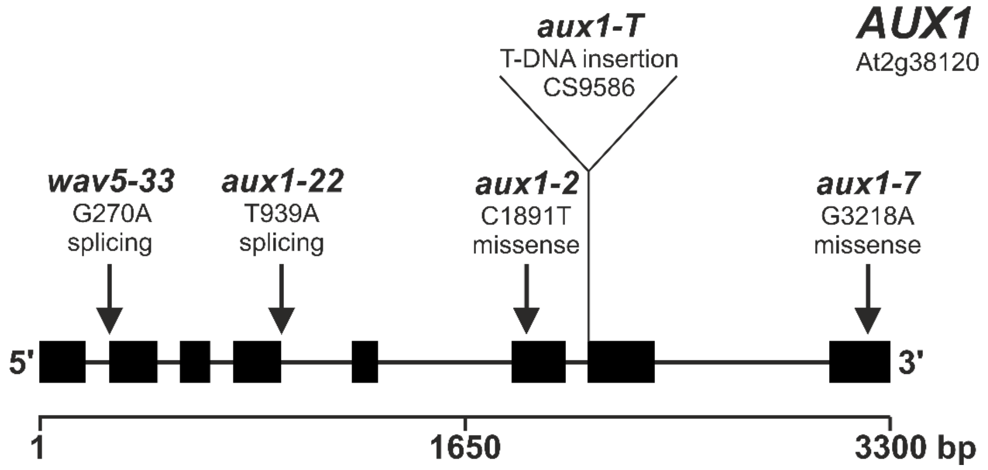

Supplementary Fig. 1. Genomic model, indicating mutations in *Arabidopsis thaliana AUX1*. The genomic model of *AUX1* is presented from the start- to the stop-codon including the true to scale positions of exons (black boxes) and introns (black lines). *AUX1*-mutants used in this study are shown with their underlying mutation and the position is indicated by the arrows.

Supplementary Figure 2

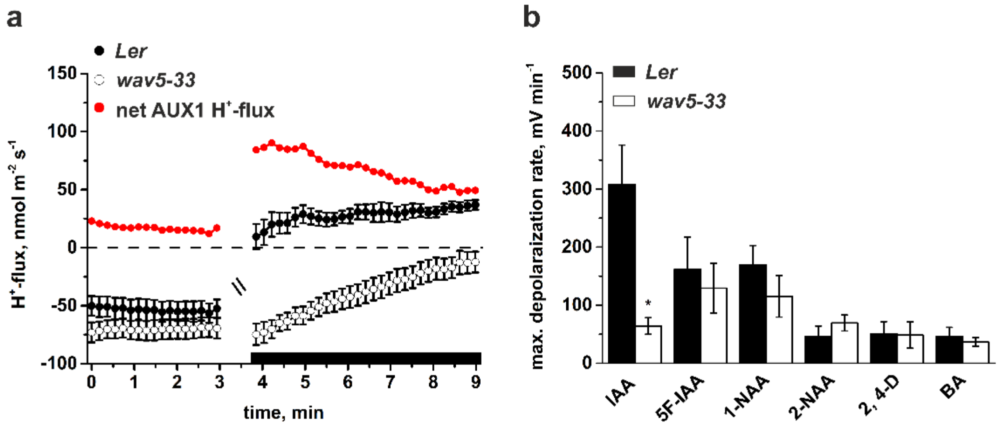

Supplementary Fig. 2. AUX1-dependent H^+-fluxes and depolarization of root hairs. **a** H^+-fluxes determined with ion selective electrodes, scanning in close proximity of root hair cells of *A. thaliana Ler* wild type (black symbols) and the AUX1-mutant *wav5-33* (open symbols), plotted against time. The red symbols depict the AUX1-specific H^+-fluxes as obtained by subtracting values of wav5-33 from *Ler*. After 3 min. of stable measurements, IAA was applied to the bath solution to a final concentration of 10 µM (black bar, curves are interrupted). (n=13 ± s.e.m. for *Ler* and n=10 ± s.e.m. for *wav5-33*). **b** Max. depolarization rates of *A. thaliana Ler* (closed bars) and *wav5-33* (open bars) root hair cells in response to 3-IAA and several auxin analogs. All auxins were applied at a concentration of 10 µM for 1 s via an application pipette. Average values are shown (n=8 ± s.e.m. for 3-IAA and n=5 ± s.e.m. for other substances). The Asterisk marks a significant difference (Students t-test, $p<0.05$)

Supplementary Figure 3

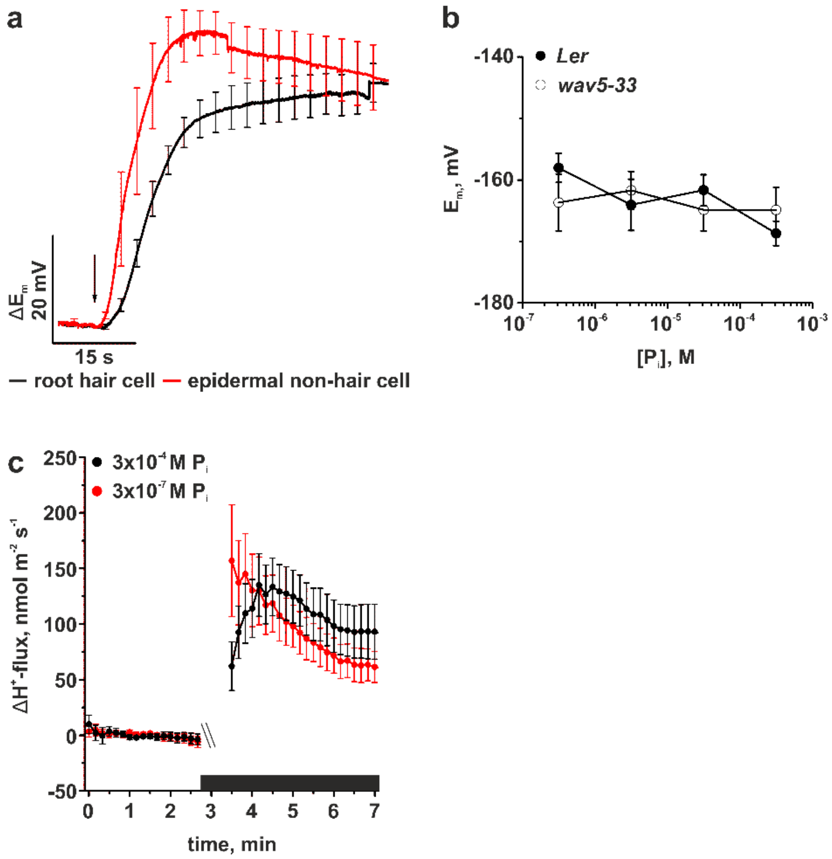

Supplementary Fig. 3. Impact of *AUX1* expression on IAA-induced depolarization in root epidermal cells. **a** IAA-dependent changes in the membrane potential of root hair (black) and non-hair epidermal root cells (red). Average traces are shown of cells stimulated with 10 µM IAA for a period of 1s (as indicated by the arrow). Error bars show s.e.m. (n=5). **b** Average membrane potential of root hairs of *A. thaliana Ler* (closed circles) and *wav5-33* (open circles), of seedlings grown under standard and phosphate-starved conditions. Error bars show s.e.m. (n=14 (*Ler*) and 9 (*wav5-33*)). **c** H^+-fluxes determined with ion selective electrodes, scanning in close proximity of root hair cells of *A. thaliana Ler* wild type, plotted against time. Seedlings were grown under standard (black) and phosphate-starved (red) conditions. After 3 min. of stable measurements, IAA was applied to the bath solution to a final concentration of 10 µM (black bar, curves are interrupted). Average values are shown. Error bars show s.e.m. (n=18 for high P_i and n=22 for low P_i).

Supplementary Figure 4

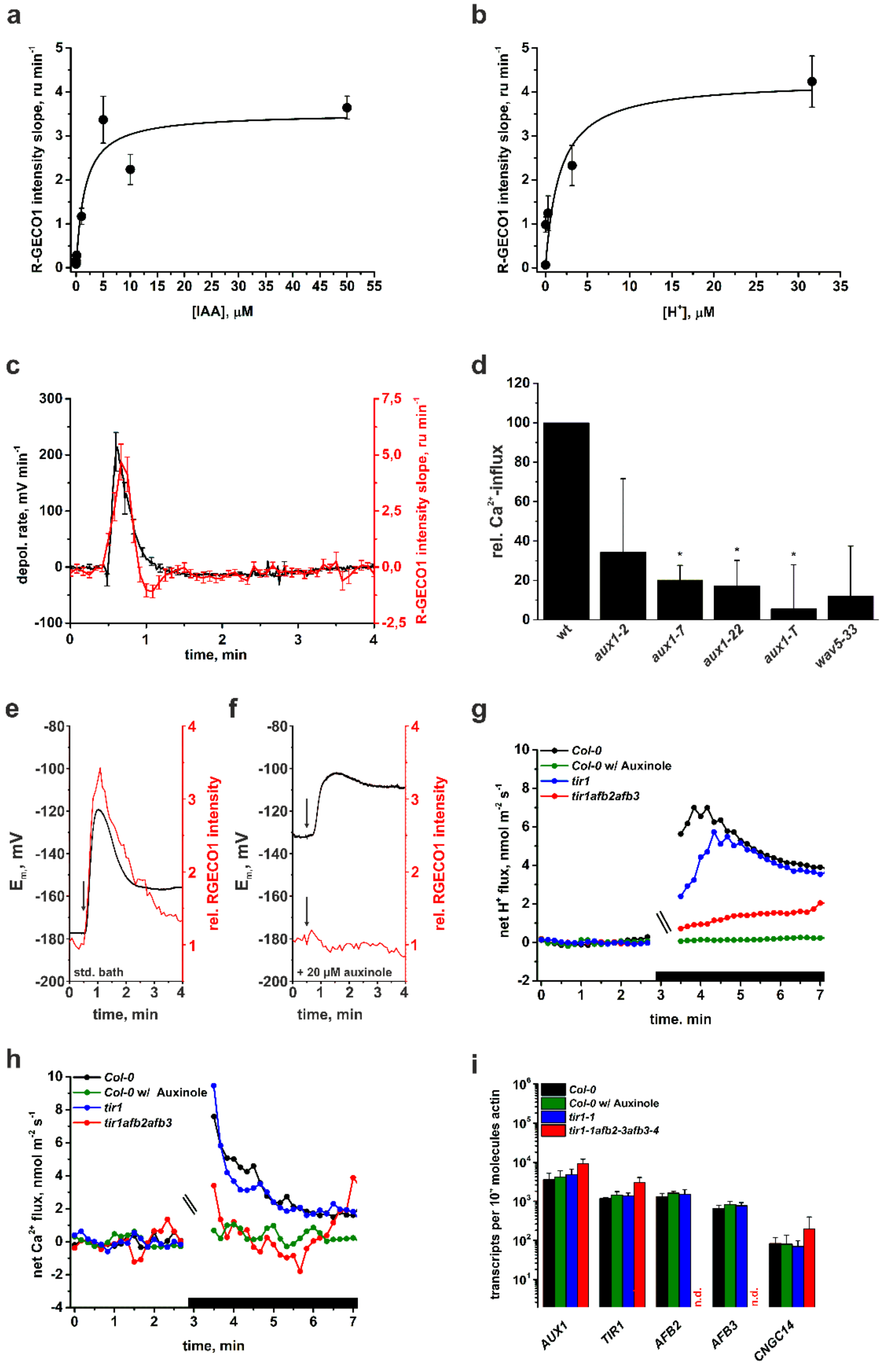

Supplementary Fig. 4. IAA-induced Ca^{2+}-signals and the role of $SCF^{TIR1/AFB}$ receptors in fast IAA responses. **a** Dose-response curve of the IAA concentration applied and the slope of changes in relative R-GECO1 fluorescence intensity, from experiments shown in Fig. 4d. A Michaelis-Menten relationship was fitted through the average data (n=7) and revealed a half maximal change of the R-GECO1 signal at an apparent IAA concentration of 1.6 µM (s.e.m.=0.9 µM). Error bars represent s.e.m. Note that the Km value was calculated from undiluted auxin concentrations inside application pipettes. **b** Change in slope of relative R-CEGO1 fluorescence intensity change as a result of application of 10 µM IAA plotted against the extracellular H^+-concentration. A Michaelis-Menten relationship was fitted through the average data (n=6) and revealed a half maximal change of the R-GECO1 signal at an H^+ concentration of 1.7 µM (s.e.m.=1.4 µM), which corresponds to a pH of 5.8. Error bars show s.e.m.. **c** Time dependent changes in slope of the IAA-induced depolarization of root hairs (black, left axis) and the slope cytosolic Ca^{2+} changes, as reported by R-GECO1 fluorescence intensity (red, right axis). Error bars show s.e.m. (n=26). **d** Average instantaneous Ca^{2+}-influx response evoked by 10 µM IAA in wild type, *aux1-2* (*Ler*), *aux1-7* (*Col-0*), *aux 1-22* (*Col-0*), *aux1-T* (*Ws*) and *wav5-33* (*Ler*). Values for mutants are given as the percentage of the response of the respective accessions (for mutants n= 12 ± s.e.m., wild type responses were measured with n=12 to 13 ± s.e.m.). Asterisks indicate significant reductions (Students t-test, $p<0.05$). **e** Representative simultaneous recordings of the PM potential (black, left axis) and the cytosolic R-GECO1 intensity of an impaled root hair cell (red, right axis), measured in standard bath solution. The arrows mark a 1 s pulse of 10 µM 3-IAA. Fluorescence values were normalized to the value just before IAA application. **f** Representative simultaneous recordings of the PM potential (black, left axis) and the cytosolic R-GECO1 intensity of an impaled root hair cell (red, right axis), measured in standard bath solution supplied with 20 µM auxinole. Arrows mark a 1 s pulse of 10 µM 3-IAA. Fluorescence values were normalized to the value just before IAA application. **g** Time course of H^+-fluxes, determined with ion-selective electrodes, scanning in close proximity of root hair cells of *A. thaliana Col-0* in the presence (green) and absence (black) of 20 µM auxinole, as well as of the *tir1-1* (blue) and *tir1-1afb2-3afb3-4* (red) mutants. After 3 min., IAA was applied to a final concentration of 10 µM (black bar, curves are interrupted at time point of IAA stimulation). Representative measurements from 4 to 16 experiments are shown. **h** Time course of Ca^{2+}-fluxes of *A. thaliana Col-0* roots in the presence (green) and absence (black) of 20 µM auxinole as well as of the *tir1-1* (blue) and *tir1-1afb2-3afb3-4* (red) mutants. IAA was applied to a final concentration of 10 µM (black bar, curves are interrupted at time point of IAA stimulation). Representative measurements from 4 to 16 experiments are shown. **i** Whole seedling expression levels of *AUX1*, *TIR1*, *AFB2/3* and *CNGC14* in *A. thaliana Col-0* in the presence (green, n=3) and absence (black, n=4) of 20 µM auxinole as well as of the *tir1-1* (blue, n=4) and *tir1-1afb2-3afb3-4* (red, n=4) mutants. Error bars show s.d. *Note that* the transcripts of AFB2 and 3 are below the detection limit in the *tir1-1afb2-3afb3-4 triple mutant and tir1-1 represents a point mutation (glycine to aspartate substitution at position 147).*

Supplementary Figure 5

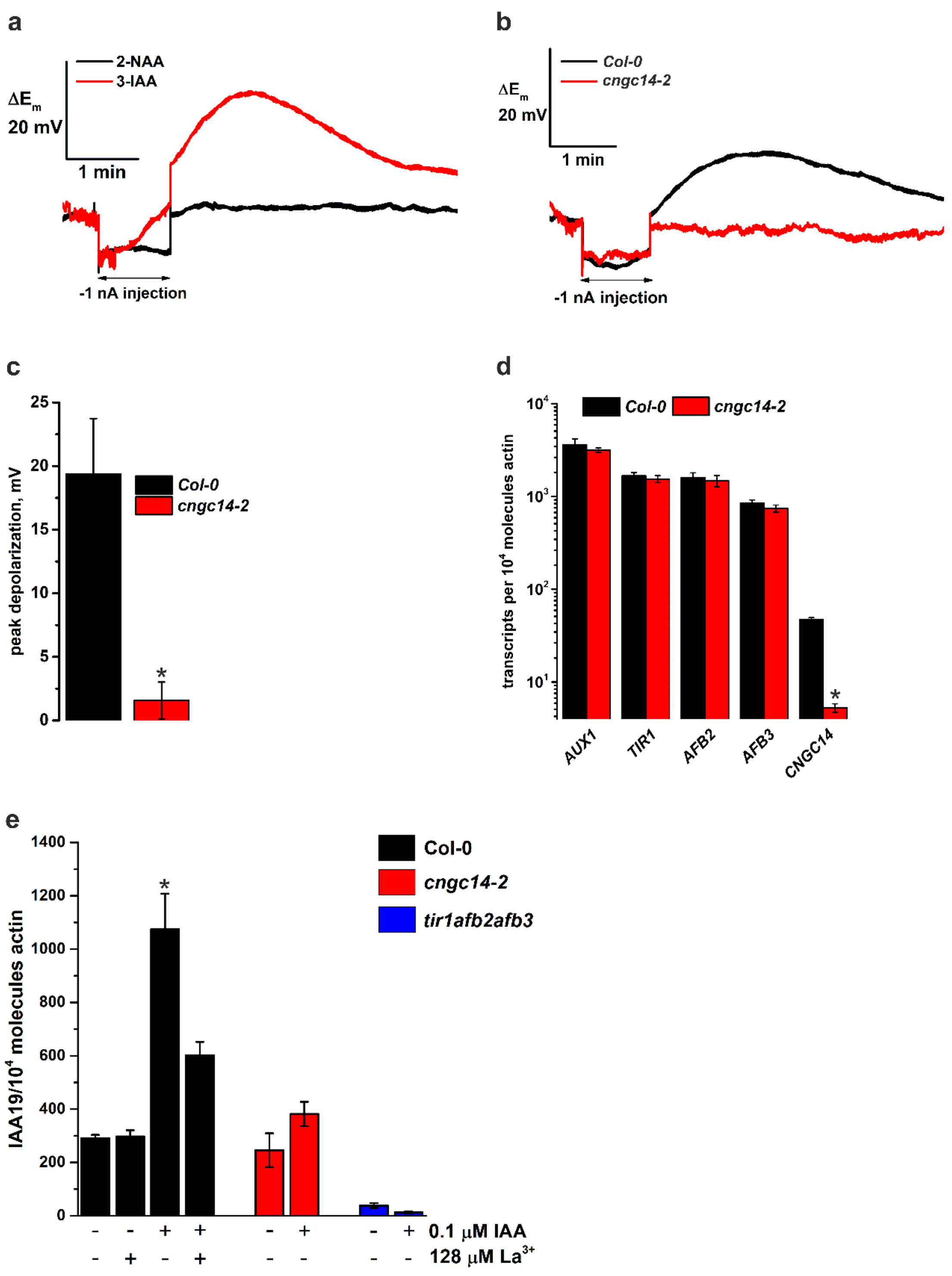

Supplementary Fig. 5. Role of CNGC14 in the IAA-induced depolarization of root hairs. **a** Depolarization of root hair cells induced by cytosolic application of auxin. Shown are representative voltage traces obtained by 3-IAA (red, from 20 experiments) and 2-NAA injection (black, from 11 experiments) via double-barreled microelectrodes. The period of iontophoretic injection of the auxins is indicated below the traces. Note that a slow depolarization follows the injection of 3-IAA, but not the stimulation with 2-NAA. **b** Membrane potential traces of *A. thaliana Col-0* (black) and *cngc14-2* (red) root hair cells stimulated by cytosolic application of 3-IAA via double-barreled microelectrodes. Shown are representative voltage traces from 7 experiments. The period of iontophoretic injection of 3-IAA is indicated below the traces. **c** Average values of max. depolarization of *A. thaliana Col-0* and *cngc14-2* root hair cells, in response to iontophoretical intracellular injection of 3-IAA. Error bars show s.e.m. (n=7) The asterisk marks a significant difference to *Col-0* (Student's t-test, $p<0.05$). **d** Whole seedling expression levels of *AUX1*, *TIR1*, *AFB2/3* and *CNGC14* in *Col-0* (black) and *cngc14-2* (red). Error bars show s.e.m. (n=5). The asterisk marks a significant difference to *Col-0* (Student's t-test, $p<0.05$). **e** Whole seedling expression levels of *IAA19* in *Col-0* (black), *cngc14-2* (red) and the *tir1afb2afb3* mutant (blue). Five days old seedlings grown on standard medium were accustomed to the standard bath for 20 min. 3-IAA was applied at 0.1 µM for 1 min followed by two washing steps. When indicated, seedlings were treated with 128 µM La^{3+} 10 min before auxin application. Seedlings were harvested for mRNA extraction 1 h after auxin or mock treatment. Error bars show s.e.m. (n=4 to 6). The asterisk marks a significant difference to mock treatment (Student's t-test, $p<0.05$). Note that La^{3+} affected auxin-induced expression of *IAA19* in wild type seedlings in a non-significant manner.

Supplementary Figure 6

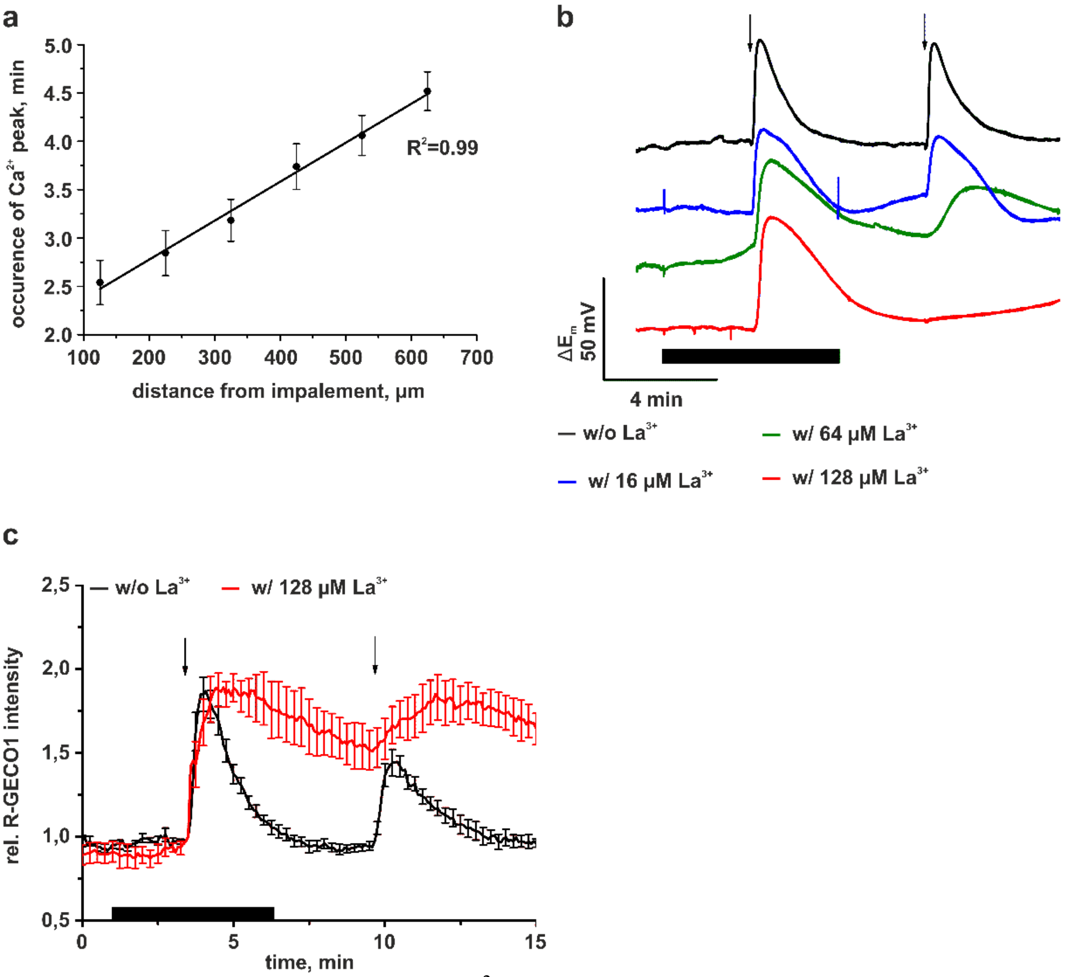

Supplementary Fig. 6. Impact of La^{3+} on IAA-induced root hair plasma membrane depolarization and cytosolic Ca^{2+} signals. **a** Propagation of the auxin injection-induced Ca^{2+} wave. Shown are the time points after the start of the auxin injection at which the peak R-GECO1 fluorescence occurred in ROIs along the root at the indicated distances from the impalement site. The time points of peak fluorescence were obtained by fitting the experimental data to a polynomial function of the ninth grade. Error bars show s.e.m. (n=7 to 8). The solid line represents linear regression (R^2=0.99). **b** Traces of root hair plasma membrane potentials of wild type seedlings. The bath solution was constantly perfused during the experiments. The black bar below the traces indicates the period of six minutes in which cells were exposed to La^{3+} in the bath solution. La^{3+} was applied at concentrations of 16 µM (blue), 64 µM (green) and 128 µM (red). The arrows indicate the period of 1 s during which 1 µM of IAA was applied from an application pipette. For clarity, the voltage traces representative of 7 (with La^{3+}) to 10 (control) experiments are presented above each other. **c** Time course of average R-GECO1 fluorescence signals in roots stimulated with two successive pulses of 1s with 1 µM IAA (as indicated by the arrows) in absence (black) and presence of 128 µM La^{3+} (red). The bath solution was constantly exchanged during the experiment. The black bar indicates the period of six minutes in which La^{3+} was present in the bath solution. Error bars show s.e.m. (n=9 for control and n=6 for La^{3+}).

Supplementary Figure 7

Consensus topology model of AUX1 with putative phosphorylation sites

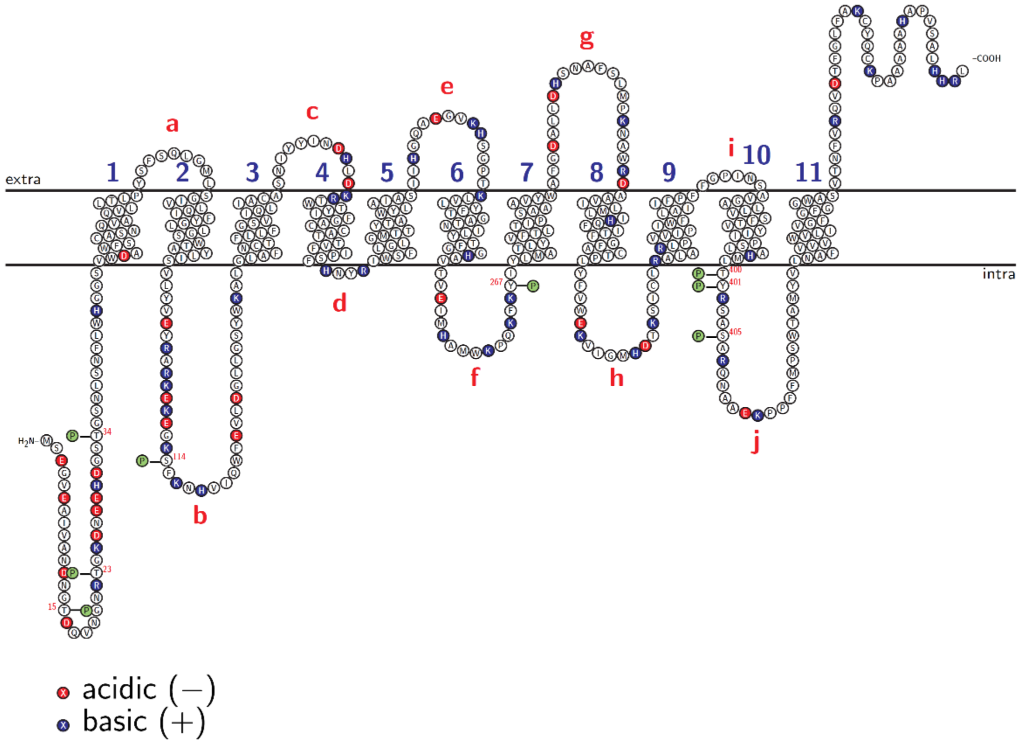

Supplementary Fig. 7. Topology model of AUX1, based on the trans-membrane consensus prediction of the Aramemnon database (http://aramemnon.uni-koeln.de). Cytoplasmic phosphorylations sites (green) are indicated as predicted by the PhosPhAt 4.0 data base (http://phosphat.uni-hohenheim.de).

Supplementary Figure 8

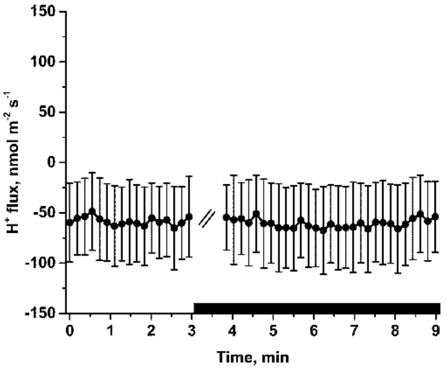

Supplementary Fig. 8. Impact of the solvent EtOH on basal H^+ fluxes at the *Arabidopsis* root. Time course of average H^+-fluxes, determined with ion-selective electrodes, scanning in close proximity of root hair cells of *A. thaliana Col-0.* EtOH was applied to a final concentration of 0.01% (v/v, black bar). The curve is interrupted at the time point of EtOH application. Error bars show s.e.m., n=4.

Supplementary Methods

Electrode preparation, calibration and experimental set-up for ion flux measurements

The electrodes were pulled from borosilicate glass capillaries w/o filament (Ø 1.0 mm, Science Products GmbH) with a vertical puller (Narishige Scientific Instrument Lab). They were baked over night at 220°C and silanized with N,N-Dimethyltrimethylsilylamine (Sigma-Aldrich) for 1 h. H^+-selective electrodes were backfilled with 40 mM KH_2PO_4/ 15 mM NaCl and tip filled with the hygrogen ionophore I cocktail A (Sigma-Aldrich). Ca^{2+}-selective electrodes were backfilled with 500 mM $CaCl_2$ and tip filled with the calcium ionophore I cocktail A (Sigma-Aldrich). Calibration of H^+-selective electrodes was performed at pH 4 and pH 7 and Ca^{2+}-selective electrodes were calibrated in solutions containing 10, 1 and 0.1 mM $CaCl_2$. Only electrodes were used that recorded a shift in voltage of approximately 59 mV per pH unit, or approximately 29 mV per pCa unit. The ion-selective electrodes were positioned with a Micromanipulator (PatchStar, Scientifica) or a SM-17 Micromanipulator (Narishige Scientific Instrument Lab) at approx. 5 µm distance to root epidermal cells of the early differentiation zone, using either an inversed microscope for recordings with single electrodes (Axiovert 135, Carl Zeiss AG) or an upright microscope (Axioskop, Carl Zeiss AG) for dual H^+/Ca^{2+} flux recordings. The electrodes were connected via Ag/AgCl half-cells to head stages of microelectrode amplifiers (custom-built for H^+ and or IPA-2, Applicable Electronics, for dual H^+/Ca^{2+} flux recordings). Electrodes were scanning at 10 s intervals over a distance of 50 or 100 µm, using a piezo stepper (Luigs & Neumann GmbH) or a micro-stepping motor driver (US Digital, USA). Raw data were acquired with a NI USB 6259 interface (National Instruments), using custom-built Labview-based software "Ion Flux Monitor". Raw voltage data were converted offline into ion flux data, as described (1, 2).

Supplementary References

1. Newman IA (2001) Ion transport in roots: measurement of fluxes using ion-selective microelectrodes to characterize transporter function. *Plant Cell Environ.* 24(1):1-14.
2. Arif I, Newman IA, & Keenlyside N (1995) Proton Flux Measurements from Tissues in Buffered Solution. *Plant Cell Environ.* 18(11):1319-1324.
